# Perioperative risk factors for postoperative pneumonia after major oral cancer surgery: A retrospective analysis of 331 cases

**DOI:** 10.1371/journal.pone.0188167

**Published:** 2017-11-14

**Authors:** Jieyun Xu, Jing Hu, Pei Yu, Weiwang Wang, Xingxue Hu, Jinsong Hou, Silian Fang, Xiqiang Liu

**Affiliations:** 1 Department of Oral Maxillofacial–Head & Neck Oncology, Guanghua School of Stomatology, Hospital of Stomatology, Sun Yat-sen University, Guangzhou, China; 2 Guangdong Provincial Key Laboratory of Stomatology, Guangzhou, China; 3 Department of Immunology and Infectious Diseases, the Forsyth Institute, Cambridge, Massachusetts, United States of America; 4 Division of General Practice and Materials Science, the Ohio State University College of Dentistry, Columbus, Ohio, United States of America; 5 Department of Oral and Maxillofacial Surgery, the Sixth Affiliated Hospital, Sun Yat-sen University, Guangzhou, China; SRI International, UNITED STATES

## Abstract

**Objective:**

Postoperative pneumonia (POP) is common and results in prolonged hospital stays, higher costs, increased morbidity and mortality. However, data on the incidence and risk factors of POP after oral and maxillofacial surgery are rare. This study aims to identify perioperative risk factors for POP after major oral cancer (OC) surgery.

**Methods:**

Perioperative data and patient records of 331 consecutive subjects were analyzed in the period of April 2014 to March 2016. We individually traced each OC patient for a period to discharge from the hospital or 45 days after surgery, whichever occur later.

**Results:**

The incidence of POP after major OC surgery with free flap construction or major OC surgery was 11.6% or 4.5%, respectively. Patient-related risk factors for POP were male sex, T stage, N stage, clinical stage and preoperative serum albumin level. Among the investigated procedure-related variables, incision grade, mandibulectomy, free flap reconstruction, tracheotomy, intraoperative blood loss, and the length of the operation were shown to be associated with the development of POP. Postoperative hospital stay was also significantly related to increased incidence of POP. Using a multivariable logistic regression model, we identified male sex, preoperative serum albumin level, operation time and postoperative hospital stay as independent risk factors for POP.

**Conclusion:**

Several perioperative risk factors can be identified that are associated with POP. At-risk oral cancer patients should be subjected to intensified postoperative pulmonary care.

## Introduction

Postoperative pneumonia (POP) is common and results in prolonged hospital stays, higher costs, increased morbidity and mortality [1]. Incidence rates for POP have been reported between 0.49% and 54.3%, depending on the type of surgery and patient population [2, 3]. POP has been shown to be associated with both patient-related and procedure-related risk factors [4, 5]. Multiple variables including advanced age, male sex, poor underlying medical condition, surgery and a higher American Society of Anesthesiologists (ASA) grade, tracheotomy and reintubation [5–10] shown to be associated with an increased risk of POP.

Major surgical procedure of advanced head and neck cancer involves radical tumor resection, lymphadenectomy, reconstruction, tracheotomy, and base of skull cancer surgery [11, 12]. In particular, microvascular free flaps are the most extensively used for large defects reconstruction in the recent past. Hence, major head and neck surgical patients may be at particularly high risk for POP, as many of the variables associated with an increased risk of POP are inherent comorbidities. Postoperative pulmonary complications rates in head and neck reconstructive patients have been variously at 7.1% to 32.7% [13–15]. The increased incidences of POP in head and neck surgery have been related to advanced ages, male gender, tracheotomy, long operative time and delayed mobilization [10, 13, 16]. However, as essential parts of head and neck cancer, a paucity of data exists regarding POP in OC patients. Similar major surgeries procedures were carried forward in OC. To our knowledge, there are no studies that have explored the role of patient- and procedure-related risk factors for POP in patients with major oral cancer surgery.

The purpose of this study was therefore to determine the incidence of POP among patients undergoing major oral cancer resection, and in particular to compare the incidence of this complication between patients with or without free flap reconstruction. Our second goal was to clarify perioperative risk factors for the development of POP.

## Materials and methods

The study design was reviewed and has been approved by the Institutional Review Board of our hospital (ERC-[2016]-24). The need for patient informed consent was waived given that the retrospective nature of the study.

### Patients

A total of 453 consecutive patients underwent major oral cancer (OC) surgery between April 2014 and March 2016 at the Hospital of Stomatology, Sun Yat-sen University, were studied. Inclusion criteria were: patient age over 18 and major oral cancer resection with or without microsurgical free flap reconstruction. One hundred and twenty-two patients were excluded based on incomplete data or because the patient did not meet inclusion criteria (S1 Fig). Records were reviewed on all the remaining 331 patients. In addition, radiography and/or chest CT scans were analyzed.

Patient-related parameters including demographic data, preoperative comorbidities (chronic obstructive pulmonary disease [COPD], diabetes, cardiovascular diseases), primary diagnosis (tumor location and UICC classification), American Society of Anesthesiologists (ASA) grade, preoperative serum albumin levels (SAL) were documented. The analyzed procedure-related parameters included incision grade, neck dissection, tracheotomy, mandibulectomy, type of flap reconstruction, time of surgery, intraoperative blood loss and postoperative days in the hospital. Analyzed parameters of the postoperative period were length of stay in hospital or 45 days after surgery, whichever occurred later.

### Diagnosis of postoperative pneumonia (POP)

POP was diagnosed based on a combination of clinical features, laboratory tests and chest X-ray or CT scan [17]. Charts were reviewed for the presence of fever (≥38°C), cough, shortness of breath and chests pain. Lab test emphasized elevated white blood cell count (>10Χ10^9^/L) and positive microbiological culture of endotracheal aspirate. Chest X-ray or CT scan demonstrated sign of inflammatory infiltrates. Blood test or X-ray was used for clinical evaluation before discharge. All equivocal results were reviewed on a case-by-case basis with an experienced pulmonologist.

### Microbiological analysis

The bacteria isolated from patients’ endotracheal aspirates were regarded as the pathogens of POP only if the counts of bacteria > 10^4^ CFU.ml^-1^ in BALF (BAL fluid) cultures or 10^5^ CFU.ml^-1^ in quantitatively endotracheal aspirate cultures (QEACs) [18]. Multidrug-resistance (MDR) of pathogens was defined as resistance to at least 3 different antibiotics [19].

### Statistical analysis

Data analysis was performed using SPSS version 22 (Chicago, IL). Univariate logistic regression analysis was performed to identify risk factors of POP. All variables with a significant value of *P* < 0.05 from univariate analysis were entered into a binary multivariate logistic regression model, which was used to identify independent predictors of POP.

## Results

Out of 331 cases included in the study, 190 (57.4%) were male and 141 (42.6%) female. The most frequent tumor entity was squamous cell carcinoma (88.5%). The most common primary sites were the tongue (42.6%), buccal mucosa (13.3%), hard palatine (7.3%), gingiva (6.9%), floor of mouth (5.1%) and oropharynx (5.1%). Neck dissection and mandibulectomy were performed in 58% and 28.7% of cases, respectively.

Reconstruction was achieved with either free flap (28.4%) or pedicle flap (17.2%). The most frequently used free flaps were anterolateral thigh free flap (15.4%) followed by radial forearm free flap (7.6%) and fibular bone free flap (5.4%). Tracheotomy (32.9%) was added to guarantee the airway of patients.

A total of 15 (4.5%) patients had POP. Of which, 11 were performed major OC surgery with free flap construction (11.6% of POP incidence). The male gender was significantly associated with POP (*P* = 0.021). Of the 15 cases developing POP, 14 (93.3%) were male. Patients with tumors on floor of mouth (*P* = 0.001), a lower preoperative SAL (*P* = 0.005), advanced T stage (*P* = 0.004), advanced clinical stage (*P* = 0.008), and lymph node metastasis (*P* = 0.018) had a significantly increased risk for POP (Table 1). Moreover, procedure-related variables including incision type (*P* = 0.007), free flap reconstruction (*P* = 0.010), mandibulectomy (*P* < 0.001), tracheotomy (*P* < 0.001), operation time (*P* < 0.001) and intraoperative blood loss (*P* = 0.003) was significantly associated with an increased risk for POP (Table 2). As expected, patients who developed POP spent a significantly longer time in the hospital after operation (22.27±8.36 vs. 11.70±6.83, OR = 1.14, *P* < 0.001).

**Table 1:** Univariate analysis of patient-related parameters.

Parameter	POP		OR	95% CI	*P* value
	Yes (n = 15)	No (n = 316)			
Age	50.5 [11.3]	54.0 [13.0]	0.98	0.94–1.02	0.314
Male	14	176	11.14	1.45–85.72	0.021
Smoking	8	98	2.56	0.89–7.14	0.393
Alcohol abuse	4	45	2.17	0.67–7.14	0.196
Underlying disease	3	67	1.08	0.30–3.92	0.911
Preoperative SAL (mg/dL)	36.3 [3.3]	41.7 [3.1]	0.79	0.67–0.94	0.005
Tumor sites					0.001
Tongue	5	136	1.140	0.30–4.34	0.848
Cheek	1	43	0.721	0.08–6.63	0.773
Floor of mouth	5	12	11.923	2.84–50.00	0.001
Others	4	125			
T stage					0.004
T1-2	7	253	0.22	0.08–0.62	
T3-4	8	63			
N stage					0.018
N0	6	223	0.28	0.1–0.8	
N1-2	9	93			
Clinical stage					0.008
1–2	3	186	0.18	0.05–0.63	
3–4	12	130			
ASA grade					0.072
1–2	10	268	0.36	0.12–1.09	
3–4	5	48			

**Table 2:** Univariate analysis of procedure-related parameters.

Parameter	POP		OR	95% CI	*P* value
	Yes (n = 15)	No (n = 316)			
Incision grade					0.007
1–2	9	275	0.22	0.08–0.66	
3–4	6	41			
Free Flap	11	84	4.08	1.41–11.76	0.010
Tracheotomy	13	96	14.28	3.33–50.00	<0.001
Neck Dissection	12	180	3.03	0.83–11.11	0.092
Mandibulectomy	12	83	11.11	3.12–33.33	<0.001
Blood Loss (L)	0.9 [0.7]	0.4 [0.5]	3.045	1.479–6.272	0.003
Operation time (h)	9.7 [2.7]	4.6 [3.6]	1.38	1.18–1.61	<0.001

To identify those perioperative parameters that independently predict POP, all the above significant parameters have been entered into a multivariate logistic regression analysis. A significant association with POP was identified for patients with male gender (*P* = 0.017), lower preoperative SAL (*P* = 0.028), longer operation time (*P* = 0.014) and longer postoperative hospital stay (*P* = 0.002) (Table 3).

**Table 3:** Independent risk factors for POP by multivariate analysis.

Parameter	*P* value	OR	95% CI
Male	0.017	16.73	1.67–167.81
Preoperative SAL (mg/dL)	0.028	0.80	0.65–0.98
Operation time (h)	0.014	1.30	1.06–1.60
Postoperative hospital stay (d)	0.002	1.14	1.05–1.24

For identification of the bacterial spectrum in POP patients, endotracheal aspirate had been performed. A total of 9 different kinds of pathogens were isolated. Predominant pathogens were further identified in 11 of the 15 POP patients (Table 4). Particularly, our drug susceptibility test showed that multidrug-resistance (MDR) of the cultured bacteria was existed in 4 out of the 11 POP patients. The most common MDR bacteria were *Klebsiella pneumoniae* (2/4, 50.0%). (Table 4).

**Table 4:** Bacterial spectrum of POP cases.

No.	Pathogenic bacteria	MDR
1	*Stenotrophomonas maltophilia*	
2	*Prevotella melaninogenica*	
	*Klebsiella pneumoniae*	
3	MRSA	(+)
4	*Klebsiella pneumonia*	(+)
	*Pseudomonas aeruginosa*	
5	*Haemophilus influenzae*	(+)
6	*Pseudomonas aeruginosa*	

## Discussion

One of the main findings of this study is that the incidence of POP after radical oral cancer resection was 4.5%. In this investigated surgical cohort with free flap reconstruction, the rate of POP was 11.6%. The risk factors associated with the development of POP in the multivariate analysis were male gender, a low preoperative SAL, longer operation time and delayed postoperative hospital stay.

About the incidence of POP or postoperative pulmonary complications after oral cancer surgery, the literature ranges between 5.1% and 30.6% [6, 10, 20, 21]. These disparities may be explained by different definitions of POP, different diagnostic methods applied and heterogeneity of surgical populations among studies. The incidence reported by us fits with the results of the comparable literature in the low range of the incidence reported.

POP is one kind of hospital-acquired pneumonia and bacteria are one of the crucial pathogens. However, literature regarding the bacteria pathogens and POP in patients with oral cancer surgery is limited. Akutsu Y et al. reported that pathogens in preoperative dental plaque are risk factors for POP following thoracotomy in patients undergoing esophagectomy [22]. In 73.3% of cases we could isolate a bacteria pathogen. Interestingly, Gram-negative bacilli were the principal microorganisms (89%). These findings coincide with previous reports [23–25] and suggest that a broad initial antimicrobial treatment, including Gram-negative coverage, is necessary when treating these pneumonias with antibiotics. Of note, as prophylactic antibiotics abused in the past decades, multidrug resistance (MDR) against bacteria pathogens is increasing. Moolchandani et al. found the prevalence of multidrug resistant Gram-negative bacilli (MDR-GNB) in hospital-acquired infections reached 55.7% [19]. In our study, 26.7% of the pathogens, including *Klebsiella pneumoniae*, MRSA and *Haemophilus influenzae* are present MDR. Hence, perioperative antimicrobial monitoring and drug susceptibility test for guiding clinical medication is indispensable.

In our study, male gender, a low preoperative SAL, longer operation time and delayed postoperative hospital stay were identified as being associated with a higher risk for POP in the multivariate analysis. Evidence from other studies suggested that male sex might be a risk factor for POP [5, 6, 26]. A proposed explanation for the finding might be that, in our patient population, smoking was significantly more often present in male (53.7% vs. 2.8%, *P* < 0.05). Of the 15 POP cases, 14 (93.3%) were male, and of the 106 smokers, 103 (97.2%) were male.

Low serum albumin level (SAL) (< 35 mg/dL), or hypoalbuminemia, is a well- documented risk factor for postoperative pulmonary complications in many patient populations [27, 28]. The ACP guidelines for preoperative pulmonary risk stratification contain good or fair evidence for low SAL being powerful predictor for postoperative complications after noncardiothoracic surgery in general [29]. In agreement with these findings, patients who developed POP had a significantly lower SAL in this cohort. Our results further confirmed that low preoperative SAL was an independent predictor for POP. In this regard, optimizing nutritional status prior to oral cancer surgery would improve the surgical outcome.

Numerous reasons can be proposed for the association between operation time and POP. First, a prolonged operation time (> 3 h) results in longer duration of anesthesia and mechanical ventilation. Maintenance of intubation anesthesia and prolonged mechanical ventilation led to abnormality of deglutition and respiration, gas exchange and atelectasis [30]. Accordingly, both type and time of anesthesia have been reported to be associated with incidence of POP, and longer mechanical ventilation was related to an increased incidence of POP [31–33]. Second, complicated procedures including lymphadenectomy and free flap reconstruction require longer operation time. To date, free flap reconstruction has become an integral part of the multidisciplinary treatment of oral cancers in most major medical centers. However, the perioperative course remains complex and postoperative pulmonary complications are common [10, 13]. Moreover, intraoperative fluid management got more complex as time prolonged, accompanied by higher risk of fluid overload and postoperative pulmonary complications [5]. As we know, blood loss is a critical indicator for fluid transfusion, and our study showed the blood patients with POP lost is more than the double of those without POP (mean, 900mL vs. 400mL, P = 0.003).

Strength of our study is that we paid special attention to avoid POP. One avoidance strategy comprises a comprehensive perioperative evaluation of the patient. Patients with one or more of the above mentioned risk factors for POP, had been identified and subjected to more careful monitoring and respiratory care. As the ACP recommended [28], postoperative respiratory care including selective use of a nasogastric tube, postural drainage, percussion and vibration, deep breathing exercises, suctioning, early mobilization and chest physiotherapy were routinely performed as effective methods for reducing POP.

A limitation of this study is its retrospective nature. All of the analyzed data were drawn from archived hospital database. Consequently, the influence of undocumented variables, e.g., BMI, type of perioperative administered antibiotics, intraoperative oxygenation index, could not be determined. Secondly, our study lack comparison between various types of anesthesia in effects on POP, since all of our major oral cancer surgeries were operated under endotracheal general anesthesia. In addition, without a priori sample size estimation might led to leave out some risk factors.

In conclusion, we can state that major oral cancer surgery with free flap reconstruction and tracheotomy is associated with a high risk for the development of POP. In our investigated cohort, male gender, low SAL and prolonged operation time have been identified as independent risk factors for POP. In order to mitigate POP and possible sequelae, patients with one or more of these risk factors should be subject to intensified monitoring and perioperative care.

## Supporting information

S1 Fig Flowchart of patients’ enrollment. The patients’ data were obtained from a single institution during a period between August 2014 and March 2016. We retrospectively reviewed the data and retrieved data of oral cancer patients (n = 453). We excluded patients with recurrent or secondary oral cancers, or those with other malignancies (n = 68). Patients with poor performance status (ECOG ≥ 3), liver cirrhosis, renal failure or poor heart or lung function, or who were unfit for flap reconstruction surgery, were also excluded to reduce bias and confounding factors (n = 50). Incomplete or missing medical records were excluded (n = 4). Finally, a total of 331 patients (190 male, 141 female) with primary oral cancer who received curative surgery and flap reconstruction were studied.
(RAR)
